# Motor Cortex Excitation/Inhibition Imbalance in Young Adults With Autism Spectrum Disorder: A MRS-TMS Approach

**DOI:** 10.3389/fpsyt.2022.860448

**Published:** 2022-04-14

**Authors:** Inês Bernardino, Ana Dionísio, Inês R. Violante, Raquel Monteiro, Miguel Castelo-Branco

**Affiliations:** ^1^Faculty of Medicine, University of Coimbra, Coimbra, Portugal; ^2^Coimbra Institute for Biomedical Imaging and Translational Research, University of Coimbra, Coimbra, Portugal; ^3^Institute of Nuclear Sciences Applied to Health, University of Coimbra, Coimbra, Portugal; ^4^School of Psychology, Faculty of Health and Medical Sciences, University of Surrey, Guildford, United Kingdom

**Keywords:** magnetic resonance spectroscopy, transcranial magnetic stimulation, autism (ASD), GABA, glutamate

## Abstract

Excitatory/inhibitory imbalance has been suggested as a neurobiological substrate of the cognitive symptomatology in Autism Spectrum Disorder (ASD). Studies using magnetic resonance spectroscopy (MRS) attempted to characterize GABA and Glutamate brain levels in ASD. However mixed findings have been reported. Here, we characterize both neurochemical and physiological aspects of GABA system in ASD by implementing a more comprehensive approach combining MRS and transcranial magnetic stimulation (TMS). A group of 16 young ASD adults and a group of 17 controls participated in this study. We employed one MRS session to assess motor cortex GABA+ and Glutamate+Glutamine (Glx) levels using MEGAPRESS and PRESS sequences, respectively. Additionally, a TMS experiment was implemented including paired-pulse (SICI, ICF and LICI), input-output curve and cortical silent period to probe cortical excitability. Our results showed a significantly increased Glx, with unchanged GABA+ levels in the ASD group compared with controls. Single TMS measures did not differ between groups, although exploratory within-group analysis showed impaired inhibition in SICI5ms, in ASD. Importantly, we observed a correlation between GABA levels and measures of the input-output TMS recruitment curve (slope and MEP amplitude) in the control group but not in ASD, as further demonstrated by direct between group comparisons. In this exploratory study, we found evidence of increased Glx levels which may contribute to ASD excitatory/inhibitory imbalance while highlighting the relevance of conducting further larger-scale studies to investigate the GABA system from complementary perspectives, using both MRS and TMS techniques.

## Introduction

Inhibitory gamma-aminobutyric acid (GABA) system dysfunction has been hypothesized to contribute to the pathophysiology of a cluster of neurodevelopmental and psychiatric disorders (1, 2). Autism spectrum disorder (ASD) is one of the conditions for which cortical excitatory-inhibitory (E-I) imbalance has been proposed as underlying etiology (3, 4), along with other pathologies with overlapping symptomatology (2, 5, 6).

Evidence from post-mortem and animal studies have strengthen this hypothesis by demonstrating altered markers of glutamatergic and GABAergic neurotransmission (7–9). Most recently, this hypothesis has been addressed in clinical studies using [1H] magnetic resonance spectroscopy (MRS), a non-invasive technique which allows *in vivo* quantification of metabolites in the brain (3, 10). Despite the steady increase of MRS studies trying to indirectly characterize GABAergic system in ASD through the quantification of MRS-derived GABA levels, the demonstration of a consistent pattern has been proved challenging. ASD is a highly heterogeneous neurodevelopmental disorder characterized by marked impairments in social interaction and communication in the presence of repetitive stereotyped behavior, sensory anomalies, and variable levels of intellectual disability (11, 12). This phenotypical variability, probably reflecting distinct neurobiological correlates, along with different methodological approaches across studies has contributed to mixed findings.

Concerning GABA system, studies in pediatric populations have shown reduced levels across several brain regions, including frontal and auditory cortices, motor and sensorimotor areas, anterior cingulate cortex, and cerebellum (13–18), while others reported comparable levels in occipital, anterior cingulate and medial prefrontal cortices (15, 19–22). In adulthood, mixed findings have been reported with some studies showing unchanged GABA levels in different brain regions: visual, auditory, and motor cortices, dorsal and medial prefrontal cortices, superior temporal sulcus and sensorimotor areas (23–28), while others revealed higher levels in the dorsal lateral prefrontal cortex (29) and lower GABA levels in the sensorimotor cortex (30) and supplementary motor area (28). Likewise, findings regarding Glx, which stands for glutamate (Glu) + glutamine (Gln), have been inconsistent, with studies revealing higher (31–33), unchanged (20, 25, 27) or lower (24, 34) levels in both children and adults with ASD when compared with typically developing controls. The link between neurochemical alterations in ASD and cognitive symptomatology has also been explored and GABA levels were associated with sensory impairments (15, 28) and behavioral measures from ASD diagnostic tools (19, 20).

Despite the substantial value that MRS studies brought to the investigation of E-I imbalance in neurodevelopmental disorders, the static measures of GABA and Glutamate levels obtained from this technique are not able to capture the dynamic process involved in cortical excitability (35). Importantly, MRS-derived GABA and Glx levels do not equate directly to GABAergic and Glutamatergic neurotransmission, respectively. Hence, the study of E-I imbalance in ASD could benefit from the inclusion of transcranial magnetic stimulation (TMS) which directly addresses inhibitory and excitatory modulation.

TMS is a non-invasive technique which allows the assessment of cortical excitability through focal brain stimulation (36). Depending on the TMS paradigm, one can tap into different neural circuits associated with both GABAergic and Glutamatergic signaling. Paired-pulse TMS (pp-TMS) is a well-established paradigm to investigate excitatory and inhibitory mechanisms, depending on the interval between the two magnetic pulses administered. Short-interval intracortical inhibition (SICI) is assumed to reflect GABA_A_ receptor-mediated neurotransmission whereas intracortical facilitation (ICF) is thought to be mediated by glutamatergic N-methyl-D-aspartate receptors and long-interval intracortical inhibition (LICI) seems to reflect inhibition mediated by GABA_B_ (37, 38). To date, few studies investigated GABAergic neurotransmission in ASD using TMS. A recent systematic review (35) reported five studies measuring motor-evoked potentials (MEP), SICI and LICI in ASD and suggested that SICI is likely to be reduced in ASD, whereas MEP and LICI were comparable between groups.

Studies exploring the relationship between TMS physiological measures of cortical excitability and metabolites levels obtained from spectroscopy have raised the question that each technique measures specific aspects of GABAergic neurotransmission (39, 40). MRS GABA levels are thought to reflect tonic instead of phasic inhibitory processes and may not be associated with synaptic activity (41), in opposition to TMS (42). Additionally, the literature also shows that interventional TMS protocols can induce GABA changes in the expected directions (43–46) suggesting that there is a link between brain metabolism and inhibition and facilitation (41). This points to the putative complementary nature of both techniques and highlights its relevance for the study of disease mechanisms. Although some studies explored the link between brain metabolites levels and measures of cortical excitability using combined MRS-TMS in healthy subjects (39, 40), little is known in disease context.

In the current study, we aimed to comprehensively investigate the E-I balance in ASD by testing the mechanistic role of GABA neurotransmission from the neurochemical and physiological points of view. We hypothesize that GABA and Glx neurotransmitters levels as well as excitability patterns are altered in ASD, in line with the prediction of expected changes in E-I. To our knowledge, this is the first study combining both MRS and TMS techniques, in the same clinical and control groups, to address the E-I imbalance hypothesis in ASD young adults. With this in mind, we believe that our findings might also inform about the complementary nature of MRS-TMS by elucidating their relationship in both health and disease.

## Materials and Methods

### Participants

Thirty-four participants were recruited, including a group of 17 young male adults with ASD and a control group with 17 typically developing (TD) male participants. One of the ASD participants was not able to collaborate in both MRI and TMS data acquisitions and was excluded from the study. As a result, 16 ASD participants and 17 TD participants were included in the analyses.

ASD participants were recruited from a database used in previous studies (47, 48) and in collaboration with local ASD associations. All ASD participants obtained positive results on the gold standard diagnostic instruments, namely parental or caregiver interview [Autism Diagnostic Interview-Revised, ADI-R (49)] and direct structured proband assessment [Autism Diagnostic Observation Schedule, ADOS (50)], and met the current diagnostic criteria for ASD as assessed by the Diagnostic and Statistical Manual of Mental Disorders [5th ed.; DSM-5 (12)]. Exclusion criteria included genetic syndrome, neurological or psychiatric comorbidities, history of traumatic brain injury, epilepsy, contraindications to MR scanning or TMS and severe learning disabilities (full-scale intellectual quotient <85). None of the participants were diagnosed with ADHD, OCD, anxiety or mood disorders. Three ASD participants were under chronic medication for ASD-related symptomatology (methylphenidate *n* = 2; risperidone *n* = 1) and were instructed to maintain the treatment as usual.

TD participants were recruited from the local community, had no history of psychiatric and/or neurological illnesses, or contraindication to MRI or TMS acquisitions and were not taking any medication.

Participants included in the study received the Portuguese version of the Wechsler Adult Intelligence Scale, WAIS-III (51) to perform intellectual quotient (IQ) assessment. Handedness was evaluated with the Edinburgh Inventory (52). Demographic and diagnostic measures are detailed in Table 1.

**Table 1:** Demographic and diagnostic data.

	**ASD (*****n*** **=** **16)**		**CTRL (*****n*** **=** **17)**	
	**Mean (S.E.)**	**Range**	**Mean (S.E.)**	**Range**
CA (years)	20.6 (0.9)	17–30	22.9 (0.8)	16–29
Education (years)*	12.4 (0.4)	11–17	15.0 (0.5)	10–17
Sex (male:female)	16:0		17:0	
Handedness (right:center)	13:3		15:2	
Full-scale IQ*	105.4 (2.7)	86–119	120.1 (2.5)#	108–129
VIQ*	104.6 (3.6)	70–126	120.9 (2.6)#	105–137
PIQ	106.8 (3.0)	91–129	114.9 (3.6)#	101–137
**ADI-R**				
Reciprocal social interactions	15.8 (1.0)	8–25		
Language/ Communication	10.4 (0.9)	3–18		
Repetitive behaviors/Interests	5.4 (0.5)	3–9		
Developmental delay	2.1 (0.5)	0–5		
**ADOS**				
Total result	9.3 (0.3)	7–12		
Communication	2.8 (0.2)	2–5		
Social interaction	6.4 (0.3)	5–8		

ASD, Autism Spectrum Disorder group; CTRL, Control group; S.E., Standard Error; CA, Chronological Age; IQ, Intelligence Quotient; VIQ, Verbal Intelligence Quotient; PIQ, Performance Intelligence Quotient; *p < 0.05; #n = 11.

ASD and control groups were matched for chronological age (*t*_(31)_ = −1.914, *p* = 0.065), handedness (*p* = 0.656), and performance IQ (*t*_(25)_ = −1.752, *p* = 0.094). Total IQ (*t*_(25)_ = −4.035, *p* = 0.000), verbal IQ (*t*_(25)_ = −3.699, *p* = 0.001) and level of education (U = 48.000, *p* = 0.001) revealed expected differences between groups given the intellectual profile described in ASD (53, 54).

The study procedures were revised and approved by the Ethics Committee of the Faculty of Medicine of the University of Coimbra and are in accordance with the Declaration of Helsinki. All participants gave verbal informed consent. Moreover, we obtained written informed consent from participants, or their parents when appropriate.

### Procedures

The study encompassed a 1-day visit in which demographic and intellectual assessment was performed followed by MRS and TMS data acquisition. Although we did not expect TMS after-effects, stimulation was always performed after MRS to avoid possible interferences on neurochemical data.

#### Proton Magnetic Resonance Spectroscopy (1H-MRS)

We acquired Magnetic Resonance Imaging (MRI) data on a Siemens MAGNETOM Trio Tim 3T (Erlangen, Germany), equipped with a 12-channel birdcage head coil, at our facilities (ICNAS/CIBIT, University of Coimbra). During the whole MRI experiment, movement was controlled by the continuous monitoring of the eyes positioning [Eyetracker: SensoMotoric Instruments (SMI), Teltow, Germany].

Structural images were obtained through a high-resolution T1-weighted 3D MPRAGE (Magnetization Prepared Rapid Acquisition Gradient Echo) sequence, with a voxel size of 1 × 1 × 1 mm^3^ (FOV, field of view = 256 × 256 mm^2^; 176 slices; TR, repetition time = 2,530 ms; TE, echo time = 3.42 ms; TI, inversion time = 1,100 ms; FA, flip angle = 7°).

**Figure 1:**
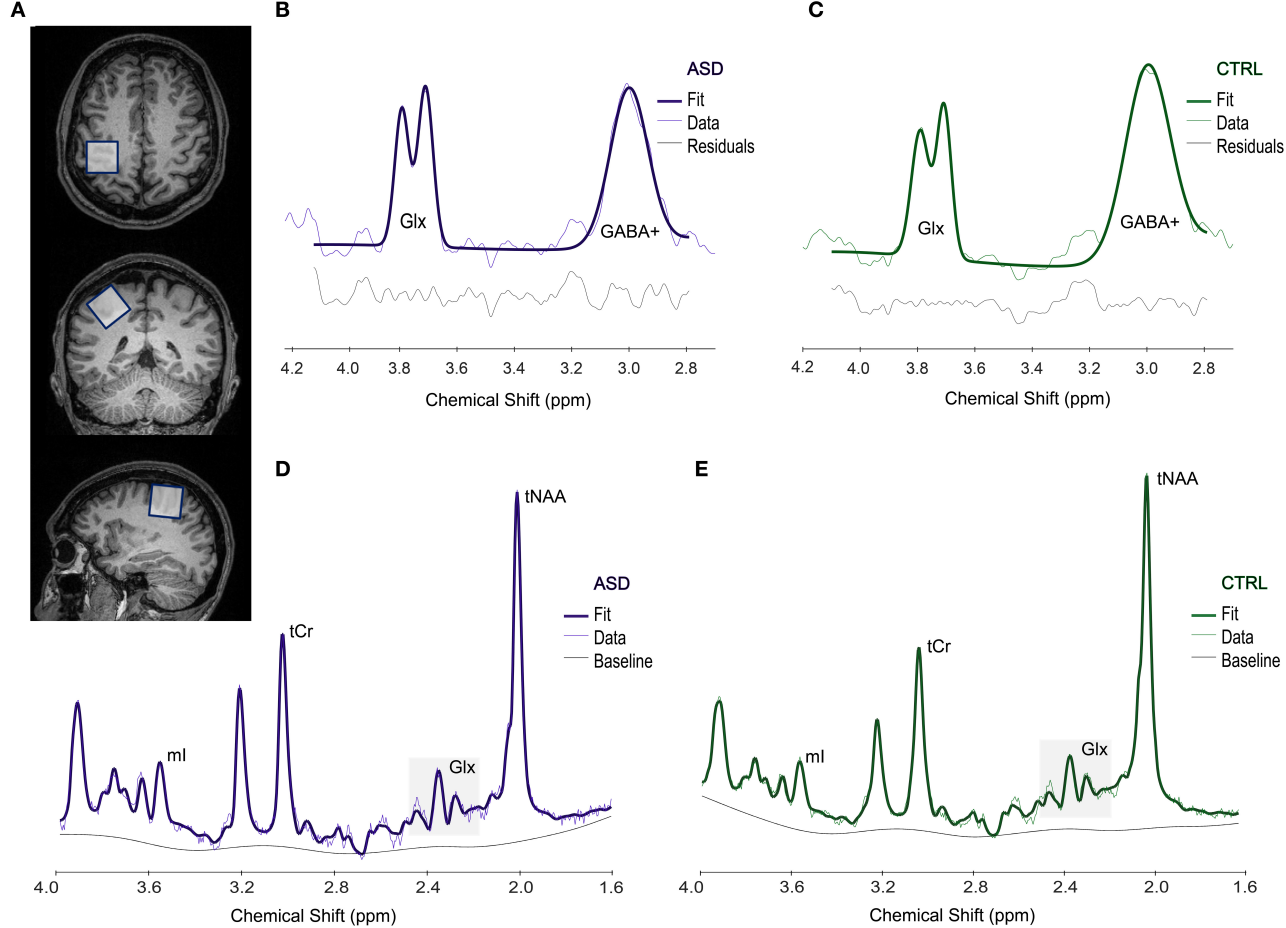
Example of the voxel placement in the motor area **(A)**. Upper panel shows a representative spectrum from an ASD **(B)** and a CTRL **(C)** participant, obtained from Gannet and used to estimate GABA+ levels. Lower panel shows a representative spectrum processed in LCModel, used to measure Glx levels in both ASD **(D)** and CTRL **(E)** groups. ASD, Autism Spectrum Disorder group; CTRL, control group; Glx, glutamate+glutamine; GABA+, gamma-aminobutyric acid; mI, myo-inositol; tCr, total creatine; tNAA, total N-acetylaspartate; ppm, parts per million.

To estimate the levels of GABA and Glx metabolites in the dominant motor cortex with both MEGA-PRESS and PRESS sequences, respectively, we first ran a functional localizer to select the motor region activated by a finger-tapping task (as in Silva et al. (55), including both synchronous and asynchronous tapping, at particular frequencies), and subsequently placed a 3 × 3 × 3 cm^3^ voxel on the corresponding location for both MEGA-PRESS and PRESS sequences. We marked the region-of-interest including the activation map and used the center coordinates of the activation to position the voxel. Small adjustments were performed to avoid including skull or the ventricles in the voxel, which could affect our data. An example of voxel positioning is presented in Figure 1.

Quality of the data was assessed following the recommended guidelines consensus (56), as detailed below.

##### GABA Quantification

The MEGA-PRESS (MEshcher-GArwood Point RESolved Spectroscopy) (57) sequence was employed (TR = 1,500 ms; TE = 68 ms; 196 averages and 1,024 points) to estimate GABA levels through a J-difference editing technique. Unsuppressed water spectra (16 averages) were also obtained in the same voxel to determine water-scaled levels. Metabolite quantification was carried out in Gannet (v. 3.1.5), a MATLAB-based GABA Analysis Toolkit (58). To improve signal-to-noise ratio (SNR), we used exponential line broadening at 3 Hz. We then processed the time-resolved data into the frequency domain with Fast Fourier Transform. We performed RobustSpecReg alignment to correct both frequency and phase to improve the quality of the spectra and applied eddy current correction to water and metabolite signals. In addition, we filtered the data with a Hankel singular value decomposition (HSVD) filter to reject residual water signal from the difference spectra. A non-linear least-squares fitting approach was used for the integration of the edited GABA (~at 3.00 ppm) through a Gaussian model applied to the difference spectrum.

We used GABA+/water levels (i.u., institutional units) and corrected the metabolite levels for voxel composition by the alpha tissue correction method (59). For this purpose, we estimated the fractions of gray matter (GM), white matter (WM) and cerebrospinal fluid (CSF) in the voxel through coregistration and segmentation by Gannet and SPM12 toolbox (Wellcome Trust Centre for Neuroimaging, Institute of Neurology, UCL, London, UK). To acknowledge a possible contribution from macromolecules and homocarnosine to GABA estimation (60), we reported our levels as GABA+. Therefore, in this work, we report corrected GABA+/water levels.

Full width at half maximum (FWHM) and SNR for the entire spectrum were determined and the overall quality of the data was evaluated by visual inspection, conducted by two researchers. Data with unsatisfactory quality or with a GABA+ fit error superior to 10% were not included for further analyses. An example of representative spectra is presented in Figure 1.

##### Glx Quantification

We applied the PRESS (Point RESolved Spectroscopy) sequence (TR = 2,000 ms; TE = 35 ms; 46 averages and 1,024 points; unsuppressed water: 16 averages) in the same location, and used LCModel v. 6.3-1M (61) to estimate Glx levels. Water scaling and eddy-current correction were performed, and spectra were obtained from 1.6 to 4.0 ppm to reduce artifacts due to the contamination from lipids and macromolecules. In this work, we present the corrected values for Glx/water, considering voxel tissue composition, namely GM, WM and CSF fractions, as fully detailed in Naaijen et al. (62). We assessed FWHM and SNR for the entire spectrum and excluded from the analyses those spectra with bad quality identified by visual inspection or with Glx Cramér-Rao Lower Bounds (CRLB) > 10%. For an example of selected spectra, please see Figure 1.

#### Transcranial Magnetic Stimulation

Transcranial magnetic stimulation was performed using a MagPro X100 magnetic stimulator (MagVenture, Denmark), equipped with a MCF-B65 figure-of-eight coil (MagVenture, Denmark). All participants wore earplugs and were resting in a comfortable armchair. The hotspot was defined as the region in the dominant primary motor cortex (M1) with the greatest response to the stimulation pulses. The coil was placed over the hotspot, tangentially to the scalp, at 45° to the sagittal plane. The electromyographic (EMG) signal was recorded in the first dorsal interosseous (FDI) muscle by Ag/AgCl electrodes (Biopac Systems, CA, USA), in a belly-tendon montage, coupled to an EMG 100C amplifier and connected to a BIOPAC MP-150 system (Biopac Systems, CA, USA), with a gain of 1,000, to register motor-evoked potentials. The Acqknowledge 4.2 software (Biopac Systems, CA, USA) was used to acquire EMG signal at a 2.5 kHz sampling rate and to process data.

##### Paired-Pulse (pp-TMS)

We first determined SI1mV, by testing different intensities until we found the minimum individual intensity that elicited motor-evoked potentials with a peak-to-peak amplitude of at least 1mV in 5 or more trials out of a total of 10 consecutive trials. For both SICI and ICF we applied a suprathreshold test pulse at 120% SI1mV, preceded by a subthreshold conditioning pulse (80% of SI1mV). In contrast, for LICI both the conditioning and test pulses were applied with an intensity of 100% SImV. We studied different protocol-dependent inter-stimulus intervals (ISIs): 1, 3 and 5 ms to study SICI; 10, 15 and 20 ms for ICF; and 50, 100 and 150 ms to assess LICI. For each protocol, we delivered 10 pairs of pulses for each ISI, in a random order, and 10 single-pulses (baseline) at the same intensity of the test stimulus, as described by De Beaumont et al. (63). We identified motor-evoked potentials and determined their peak-to-peak amplitude using an in-house script. LICI measures were excluded from the analyses because the substantial number of missing values hindered an adequate statistical comparison between groups. All measurements were validated by visual inspection, trial-by-trial. Furthermore, for each individual, we normalized the amplitudes obtained for each ISI, by calculating paired-pulse:baseline MEP amplitude ratios.

##### Input-Output or Recruitment Curve (I-O Curve)

Resting motor threshold (rMT) was defined as the lowest intensity that elicited at least 5 MEPs with peak-to-peak amplitude ≥ 50 μV out of 10 consecutive single-pulses. We constructed an input-output curve for each participant, using the following intensities: 90, 100, 110, 120, 130, and 140% of the individual rMT, as reported by De Beaumont et al. (63). Sixty pulses (10 per intensity) were applied in a randomized order. We determined the maximal peak-to-peak amplitude of MEPs, the stimulation intensity required to elicit a half-maximal MEP (S50) and the curve slope.

##### Cortical Silent Period (CSP)

The resting motor threshold was also used to select the intensity for the cortical silent period protocol. We delivered to the dominant M1 a single-pulse at 130% of rMT, in the middle of a 10-s voluntary contraction of the contralateral hand, at 20% of the participant's maximal force. The force was controlled online by the participant and an investigator, through the inspection of a hand-held digital dynamometer. This procedure was repeated for 10 trials, with an inter-trial interval of 10 s included to avoid fatigue and its potential effects in intracortical GABA_B_-mediated inhibition (64). We studied relative and absolute silent period durations, measured by two authors, with the onsets and offsets being defined according to the work from Säisänen et al. (65). When present, the breakthrough EMG activity was counted as part of the CSP and included in the measurement (66). Additionally, CSP:MEP ratios were calculated to reduce the interindividual variability intrinsic to CSP durations (66).

### Statistical Analyses

We conducted statistical treatment of all data in the SPSS Statistics software (version 27; IBM SPSS Statistics, IBM Corporation, Chicago, IL), and established a significance level of 0.05. Data normality was evaluated with the Shapiro-Wilk test and extreme outlier values excluded. We ran independent samples *t*-test for between-groups comparisons or its non-parametric equivalent Mann-Whitney U, when appropriate, reporting the exact *p*-values. Welch's *t*-test was reported wherein sample size was distinct between groups (IQ measures). Handedness was compared between groups with the Fisher's exact test. Within-group exploratory analysis performed for the paired-pulse TMS paradigm was carried out by applying the Wilcoxon test, since data were not normally distributed. Benjamini-Hochberg false discovery rate (FDR) method (67) was used to correct for multiple comparisons (α = 0.05).

In an exploratory approach, we investigated the correlations between ADI-R (Reciprocal Social Interaction, Language Communication and Repetitive Behaviors indices)/ADOS (Communication and Interaction indices) and MRS measures (GABA+ and Glx) for the ASD group, with Spearman's rho. The same test was applied to investigate possible correlations between TMS (input-output curve: slope, S50 and max MEP; ppTMS: SICI and ICF measures; CSP: absolute and relative) and MRS (GABA+ and Glx) measures, for both the ASD and CTRL groups. In those cases, wherein we found significant correlations only for one group (CTRL), we provide a comparison of effects, by testing the slope of the regression line and assuming that in the group that did not reach significance for the studied correlations (ASD) the slope is null. Given the exploratory nature of the correlation analyses, we did not correct for multiple comparisons.

## Results

### Proton Magnetic Resonance Spectroscopy (1H-MRS)

**Figure 2:**
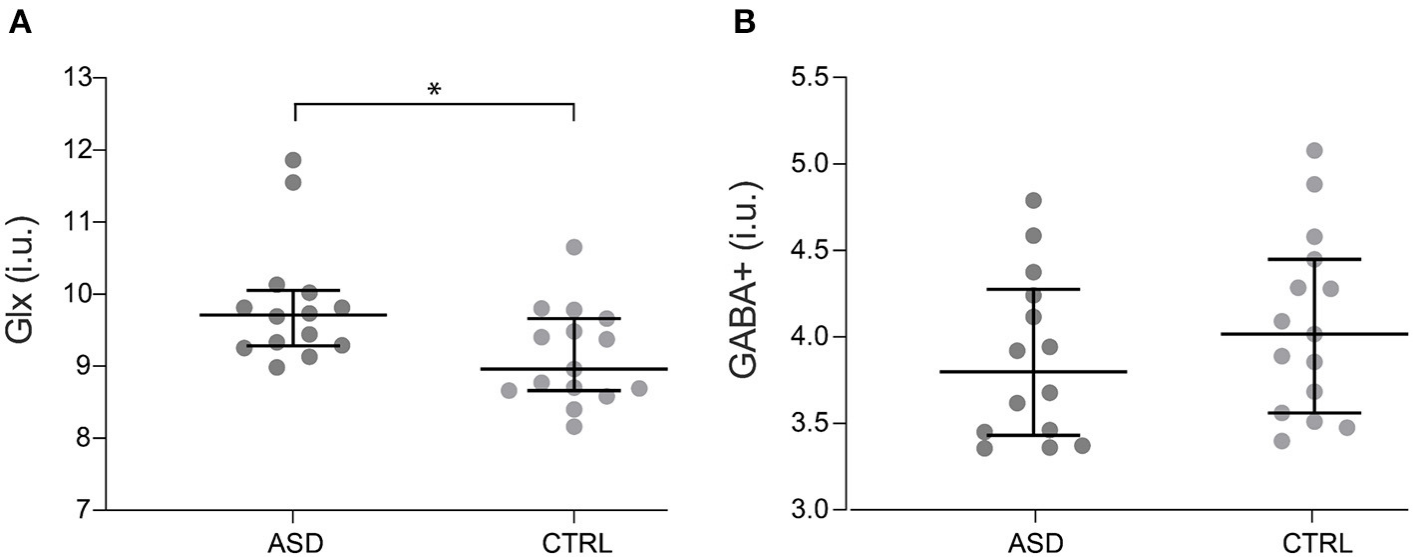
Glx **(A)** and GABA+ **(B)** levels for ASD (*n* = 14) and control participants (*n* = 15). Dots represent individual values and horizontal lines depict median and 95% confidence interval. *p < 0.05. ASD, Autism Spectrum Disorder group; CTRL, control group; GABA+, gamma-aminobutyric acid; Glx, glutamate+glutamine.

Concerning metabolite levels, tissue-corrected Glx levels in the motor cortex were significantly increased in ASD when compared to control participants, with differences surviving FDR correction (*Z* = −2.400, *p* = 0.016, Figure 2A), while corrected GABA+ levels (*t*_(27)_ = −1.032, *p* = 0.311, Figure 2B) did not show significant differences between groups.

In addition, Glx levels in patients correlated with both the Social Interaction score from ADOS (rs = −0.569, *p* = 0.034) and ADI-R Repetitive Behaviors/Interests component (rs = 0.601, *p* = 0.023). We did not find significant correlations between IQ scores and any of the MRS measures explored in the study (*p* > 0.05), which ruled out an effect of IQ in our results.

The quality of the spectra included in the analyses was partially ensured by maintaining GABA+ Fit Error and Glx Cramér-Rao values below 10% for MEGA-PRESS and PRESS sequences, respectively (Table 2). Additionally, FWHM linewidth (MEGA-PRESS: *t*_(27)_ = −0.032, *p* = 0.974 and PRESS: Z = −0.628, *p* = 0.543) and SNR (MEGA-PRESS: *t*_(27)_ = −0.272, *p* = 0.788 and PRESS: *t*_(28)_ = 0.383, *p* = 0.705) indices were equivalent between groups.

**Table 2:** MRS data quality parameters.

	**ASD (*****n*** **=** **14)**		**CTRL (*****n*** **=** **15)**	
	**Mean (S.E.)**	**Range**	**Mean (S.E.)**	**Range**
**MEGA-PRESS**				
GABA+ Fit Error (%)	5.04 (0.28)	3.82–6.90	5.19 (0.40)	2.74–8.42
FWHM (Hz)	19.17 (0.33)	16.26–20.80	19.19 (0.52)	16.94–24.47
SNR	13.52 (1.25)	6.42–20.84	13.95 (0.99)	8.21–21.88
**PRESS**				
Glx CRLB (%)	6.50 (0.20)	5–7	7.13 (0.26)	6–9
FWHM (Hz)	4.51 (0.15)	3.70–5.49	4.62 (0.11)	4.22–5.49
SNR	31 (0.74)	28–37	30.44 (1.21)	21–38

*ASD, Autism Spectrum Disorder group; CTRL, Control group; S.E., Standard Error; FWHM, Full Width at Half Maximum; SNR, Signal to Noise Ratio; CRLB, Cramér-Rao Lower Bound. MEGA-PRESS and PRESS quality data were obtained from Gannet and LcModel softwares, respectively*.

Voxel tissue composition was similar between ASD and control groups (GM: *t*_(27)_ = 0.797, *p* = 0.432; WM: *t*_(27)_ = −0.952, *p* = 0.350; CSF: *t*_(27)_ = 0.471, *p* = 0.642), excluding the impact of tissue composition in the metabolite levels explored in this study (Table 3).

**Table 3:** MRS voxel tissue proportions.

	**ASD (*****n*** **=** **14)**		**CTRL (*****n*** **=** **15)**	
	**Mean (S.E.)**	**Range**	**Mean (S.E.)**	**Range**
GM	0.33(0.005)	0.30–0.37	0.32 (0.005)	0.29–0.37
WM	0.61 (0.006)	0.57–0.66	0.62 (0.007)	0.58–0.66
CSF	0.07 (0.005)	0.03–0.11	0.06 (0.004)	0.04–0.09

*ASD, Autism Spectrum Disorder group; CTRL, Control group; S.E., Standard Error; GM, Gray Matter; WM, White Matter; CSF, Cerebrospinal Fluid*.

### Transcranial Magnetic Stimulation

**Figure 3:**
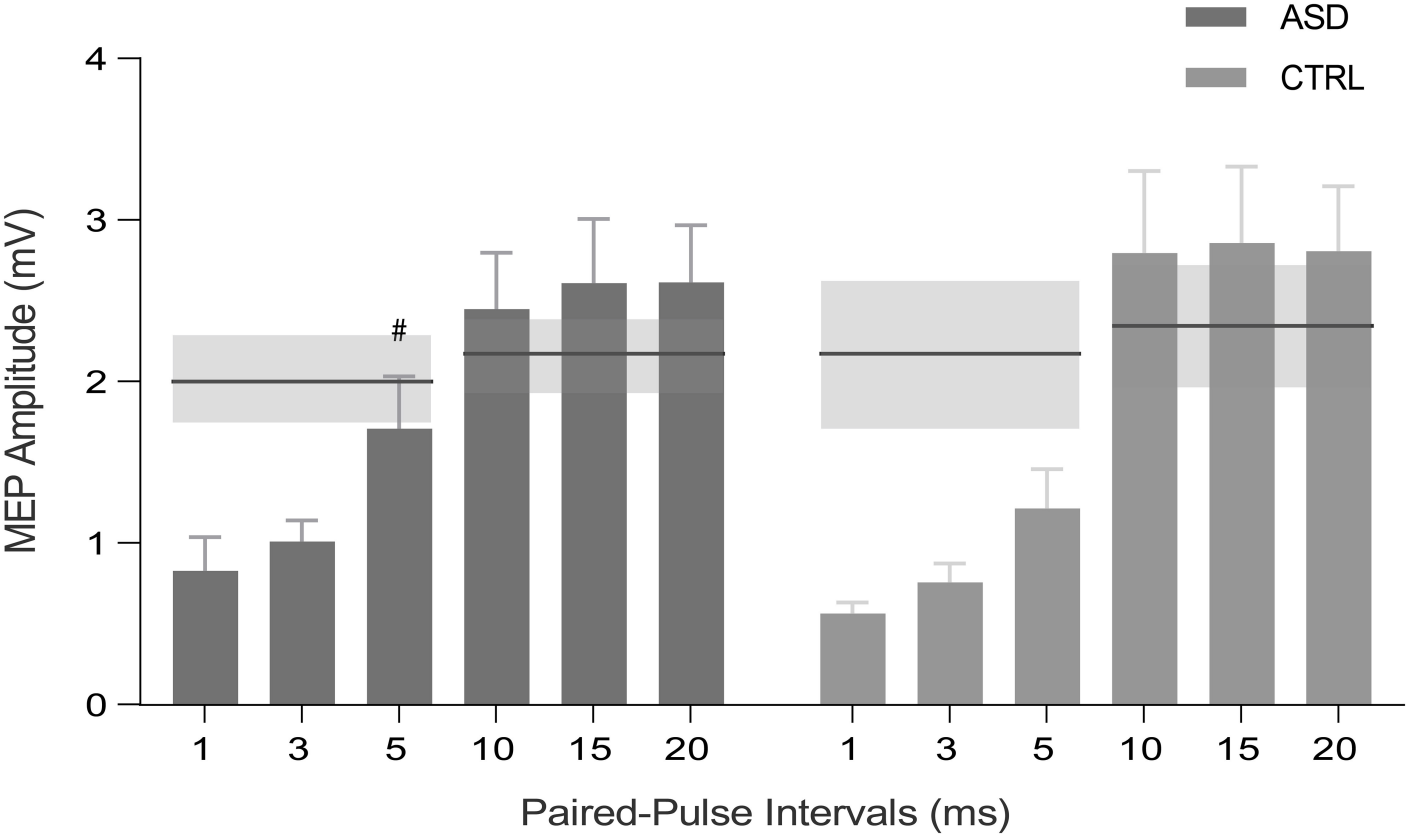
MEP peak-to-peak amplitude for SICI (1, 3, and 5 ms) and ICF (10, 15, and 20 ms) intervals for ASD and control participants (CTRL). Horizontal lines represent the baseline condition (with no inhibitory or facilitatory effects), obtained by single-pulse TMS for each group and protocol. Inhibition occurs for bars below the horizontal lines, whereas excitation stands above the lines. SE of the means are illustrated in shaded horizontal bands and error bars. #*p* > 0.05 ASD, Autism Spectrum Disorder group; CTRL, control group; MEP, motor-evoked potential; SICI, short-interval intracortical inhibition; ICF, intracortical facilitation; S.E.; standard error of the mean.

Concerning the paired-pulse protocol, within-subject analysis, where the mean MEP peak-to-peak amplitude of the conditioned stimulus was compared to the mean peak-to-peak amplitude of baseline pulses, revealed that all interstimulus intervals (ISIs) of SICI and ICF induced the expected significant inhibition and facilitation, respectively, in the control group (*p* < 0.05, with the results surviving FDR correction). Interestingly, the same effect was not verified for the ASD group at 5 ms ISI (*Z* = −1.156, *p* = 0.278) (Figure 3). Between-group differences were not observed (*p* >0.05). The other single TMS measures were not informative.

### Multimodal Correlations

Since TMS and MRS are both used in the study of cortical excitability, we investigated for a multimodal correlation between these measures.

**Figure 4:**
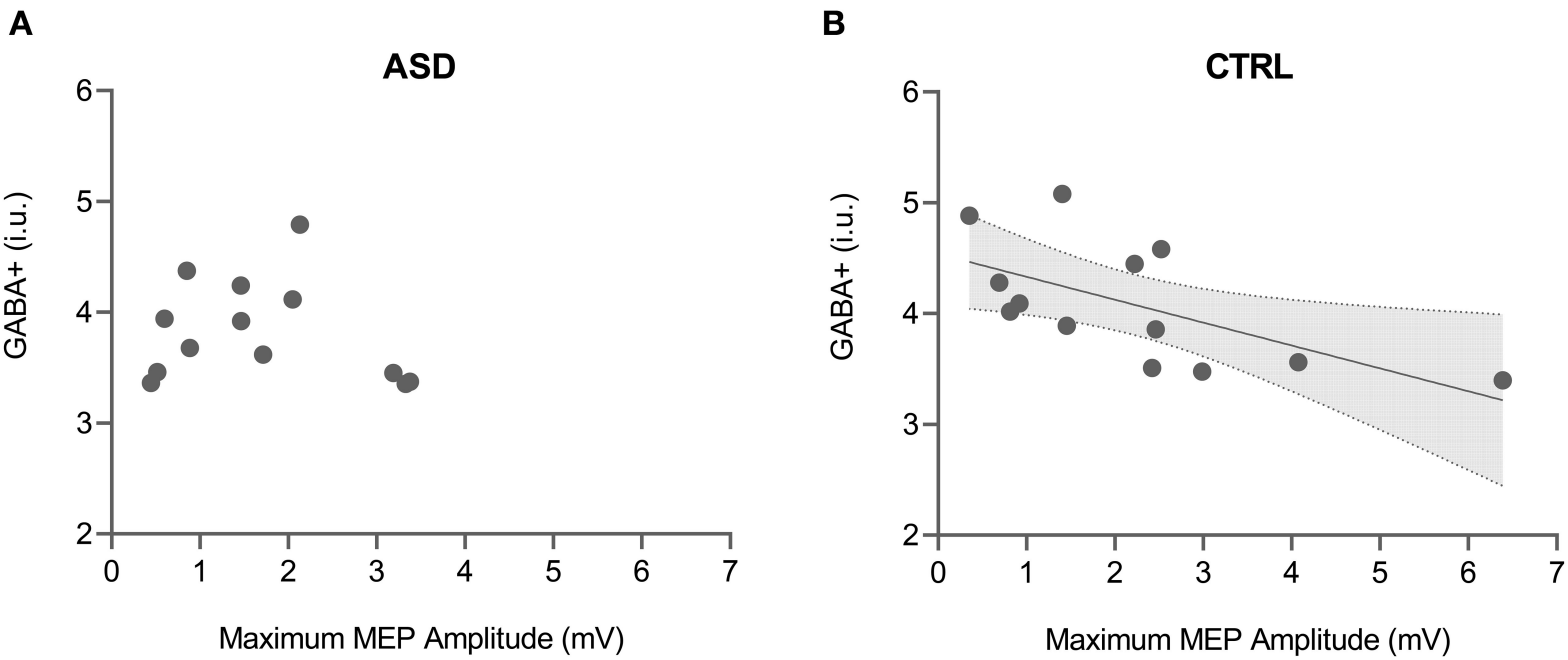
Correlation between GABA+ levels and maximum MEP amplitude from input-output TMS protocol for both ASD **(A)** and control **(B)** groups. Shaded area in the scatter plot represents 95% CI for the significant correlation. ASD, Autism Spectrum Disorder group; CTRL, control group; GABA+, gamma-aminobutyric acid; MEP, motor-evoked potential.

Both maximum MEP amplitude and curve slope of the input-output curve were found to be significantly correlated with GABA+ levels in the control group (max MEP: rs = −0.665, *p* = 0.013; curve slope: rs = −0.692, *p* = 0.009) but not in the ASD group (max MEP: rs = −0.165, *p* = 0.590; curve slope: rs = −0.148, *p* = 0.629) (Figure 4). The direct comparison of the slope of regression between groups revealed significant differences (max MEP: *p* = 0.024; curve slope: *p* = 0.032).

A significant correlation between the MEP amplitude ratio for SICI 1 ms interval and Glx levels was also found only in the control group (CTRL: rs = 0.636, *p* = 0.035; ASD: rs = −0.167, *p* = 0.693), even though the direct comparison between the regression slopes did not show significant differences (*p* = 0.086).

## Discussion

The current study aimed to comprehensively characterize GABAergic dysfunction in ASD from the neurochemical and physiological points of view, by obtaining measures from both MRS and TMS techniques.

Our findings show an increase of Glx levels in the motor cortex of individuals with ASD along with unchanged GABA+ levels. Moreover, we found a different pattern of inhibition in the ASD group for the SICI 5 ms interval.

Regarding MRS, evidence of enhanced Glx levels in ASD adults have been reported in different brain regions, such as the amygdala-hippocampal complex and auditory cortex (32, 33). In children, the same pattern was found in the anterior cingulate cortex and putamen (31, 68). These findings reinforced a long-standing hypothesis of increased excitation in ASD based on the observation of high comorbidity with seizure disorders, namely epilepsy (4). So far, few studies assessed Glx levels in motor areas in ASD. In a recent study, Kolodny et al. (27) reported non-significant differences between adults with ASD and controls in a sensorimotor region which contrasts with our findings of significantly increased Glx levels in ASD. The fact that only a partial overlap exists in voxel placement in both studies may account for these differences since there is ample evidence for metabolite's levels to be region-dependent in both human (5, 6, 69, 70) and animal (71) studies.

In which concerns GABA+ quantification, the lack of significant differences between ASD and controls observed in our study is consistent with previous research exploring E-I imbalance in adults with ASD. Although some studies reported altered GABA+ levels toward both increase and decrease (28–30), others reported null results across a wide range of areas: dorsal lateral prefrontal cortex and basal ganglia (23, 72), medial prefrontal cortex (24, 25), occipital, auditory and parietal cortices (27) and left sensorimotor cortex and left ventral premotor cortex (28). Taken together, these findings seem to indicate that GABA+ levels are not consistently altered in ASD adults. This however, contrasts with the literature in children with ASD which, despite being more inconsistent, tends to report evidence toward a decrease in the levels of this inhibitory neurotransmitter (13, 14, 17). Porges et al. (73) posited, in a meta-analysis, that GABA levels rapidly increase during development, stabilize during early adulthood, and gradually decrease during adulthood and aging. We may speculate that, in ASD, GABAergic function develops more slowly during childhood until it reaches typical levels in adulthood.

Although it was not a primary objective of this study, we explored the link between MRS measures and autistic symptomatology assessed by ADOS and ADI-R diagnostic tools since there was previous evidence, from our group, of a negative association between GABA+ and communication and developmental delay ADI-R measures in children with ASD (20). In this work, we found that, in adults, Glx levels in the motor cortex were positively correlated with repetitive behaviors and interests, measured by ADI-R, and inversely correlated with social interaction score of ADOS. The interpretation of these correlations turns out to be challenging and should be taken with caution due to their exploratory nature, but it seems to indicate that perturbed E-I balance could, at some extent, help to explain the cognitive symptomatology in ASD.

In what concerns cortical excitability, as assessed by paired-pulse TMS protocols, an exploratory within-subject analyses showed the expected pattern of significant inhibition in all the studied SICI intervals for the controls. However, for the ASD group, we did not detect a significant inhibition for the SICI 5 ms interval. The fact that the paired-pulse intervals relate to different biological processes may explain the specificity of our findings. SICI is thought to be mediated by GABA_A_ receptors which have been shown to exhibit decreased density in ASD as well as reduced protein expression for some GABA_A_ receptor subunits (74, 75). Thus, we suggest that our observation is related to alterations in the cortical inhibition mediated by GABA_A_ receptors. Alternatively, we may speculate that the transition between inhibitory (SICI) and facilitatory (ICF) intervals could be somewhat anticipated in ASD, along with the enhanced levels of excitatory Glx neurotransmitter reported in this study.

The fact that paired-pulse measures are similar between the ASD and the CTRL is in line with the work from Enticott et al. (37), who found no changes in cortical inhibition, assessed by SICI and LICI, in ASD, although showing SICI inhibition deficits in a subgroup of ASD patients who had language delay. However, in a previous study from the same authors, they reported reduced cortical inhibition (SICI) in high-functioning autism when compared to Asperger and typically developing groups (76), which may suggest that GABAergic dysfunction could be present in some ASD sub-groups characterized by specific phenotypic manifestations, including language delay (37). Although we selected a relatively homogeneous sample in what concerns to age range, intellectual functioning and diagnostic characterization, developmental acquisitions were variable in our ASD group. Given the high heterogeneity of the ASD phenotype, further stratified studies exploring different sub-groups could help to unravel the physiological specificities of E-I imbalance in this disorder. Oberman et al. (77) reported absence of changes in cortical excitability in ASD, measured by TMS, while pointing out a greater variability in the ASD group, with some participants exhibiting increased MEP amplitudes in the SICI and LICI inhibitory protocols. This agrees with our observation for the SICI 5 ms interval in the ASD group.

Regarding multimodal correlations, few studies reported associations between MRS and TMS, namely a relationship between GABA levels and SICI1ms and also slope of the TMS I-O curve (40, 41, 78). In our study, we found that GABA+ was negatively correlated with the maximum MEP amplitude and curve slope from the input-output protocol in the control group. This result was predictable from a physiological perspective since lower slope and MEP amplitudes may both be related to increased cortical inhibition (41) and refuted the opposite counterintuitive pattern observed by Stagg and colleagues (40). Remarkably, this correlation was not observed in the ASD group, as further demonstrated by direct comparisons, which suggests that the interaction between the neurochemistry and the neurophysiology underlying GABA transmission is distinct in autism. The link between these measures requires further investigation.

Further, we observed a correlation in the control group, showing that higher MEP amplitude ratio for SICI1 ms (less inhibition) was correlated with higher levels of Glx (more facilitation). To understand this relationship it is important to take into account that less inhibition can be equated with increased facilitation. Our result might therefore potentially reflect the relationship between inhibitory and excitatory mechanisms.

Here, we provided evidence supporting the E-I imbalance hypothesis in a group of adults with ASD. It is, however, important to take some limitations into consideration when interpreting our results. MRS technique provides an indirect measure of the GABAergic system which renders caution when drawing inferences from altered metabolite's quantifications. Additionally, a large voxel size was selected in order to maximize SNR leading to potential partial volumes effects (we did nevertheless correct for voxel tissue composition). Moreover, we explored specifically the motor cortex which hampers the generalization of our findings to other brain regions. In the same line, to obtain a more homogeneous group, we selected only male high-functioning individuals with ASD which does not allow us to conclude about other specific sub-groups in the spectrum. Despite the great effort in recruitment, the implementation of rigorous criteria for data quality reduced the amount of eligible data for analyses giving them an exploratory nature that should be addressed in future confirmatory studies.

This study adopted a cutting-edge approach with the aim of probing E-I imbalance hypothesis in ASD by combining MRS and TMS measures. We gathered evidence that reinforces the notion of an altered balance between excitation and inhibition hypothesis driven by increased Glx levels in ASD. Moreover, our study gives important clues into the relevance of a multimodal approach allowing for direct comparison of neurochemical (GABA and Glx) and neurophysiological outcome measures related to inhibition, such as SICI5 ms and input-output curve parameters, in the autism spectrum disorder.

## Data Availability Statement

The raw data supporting the conclusions of this article will be made available by the authors, without undue reservation.

## Ethics Statement

The studies involving human participants were reviewed and approved by Comissão de Ética da Universidade de Coimbra. The patients/participants, or their parents when appropriate, provided their written informed consent to participate in this study.

## Author Contributions

IB, IRV, and MC-B conceived the study. IB, AD, and MC-B made a substantial contribution to the study design, optimization of the protocol, and performed data analysis and interpretation. IB, AD, and RM collected data. IB wrote the first draft of the manuscript. All co-authors revised the work critically for important intellectual content and approved the manuscript in its final form.

## Funding

This research was supported by the Portuguese Foundation for Science and Technology (individual scholarship: SFRH/BPD/101641/2014 to IB and FCT/UID/4950/2020, DSAIPA/DS/0041/2020) and by the Luso-American Foundation (Prémio FLAD Life Sciences 2020). IRV is supported by the BBSRC (BB/S008314/1).

## Conflict of Interest

The authors declare that the research was conducted in the absence of any commercial or financial relationships that could be construed as a potential conflict of interest.

## Publisher's Note

All claims expressed in this article are solely those of the authors and do not necessarily represent those of their affiliated organizations, or those of the publisher, the editors and the reviewers. Any product that may be evaluated in this article, or claim that may be made by its manufacturer, is not guaranteed or endorsed by the publisher.
